# Recent Advances Regarding the Phytochemical and Therapeutic Uses of *Populus nigra* L. Buds

**DOI:** 10.3390/plants9111464

**Published:** 2020-10-29

**Authors:** Brigitta Kis, Stefana Avram, Ioana Zinuca Pavel, Adelina Lombrea, Valentina Buda, Cristina Adriana Dehelean, Codruta Soica, Mukerrem Betul Yerer, Florina Bojin, Roxana Folescu, Corina Danciu

**Affiliations:** 1 Department of Pharmacognosy, Victor Babes University of Medicine and Pharmacy, Eftimie Murgu Square, No.2, 300041 Timisoara, Romania; kis.brigitta@umft.ro (B.K.); stefana.avram@umft.ro (S.A.); ioanaz.pavel@umft.ro (I.Z.P.); lombrea.adelina@yahoo.com (A.L.); corina.danciu@umft.ro (C.D.); 2 Department of Pharmacology and Clinical Pharmacy, Victor Babes University of Medicine and Pharmacy, Eftimie Murgu Square, No.2, 300041 Timisoara, Romania; 3 Department of Toxicology, Victor Babes University of Medicine and Pharmacy, Eftimie Murgu Square, No.2, 300041 Timisoara, Romania; cadehelean@umft.ro; 4 Department of Pharmaceutical Chemistry, Victor Babes University of Medicine and Pharmacy, Eftimie Murgu Square, No.2, 300041 Timisoara, Romania; codrutasoica@umft.ro; 5 Department of Pharmacology, Faculty of Pharmacy, Erciyes University, Melikgazi, 38039 Kayseri, Turkey; mbyerer@erciyes.edu.tr; 6 Department of Functional Sciences, Victor Babeş University of Medicine and Pharmacy, 2, Eftimie Murgu Square, 300041 Timişoara, Romania; florinabojin@umft.ro; 7 Department of Anatomy and Embryology, University of Medicine and Pharmacy Victor Babeş, Eftimie Murgu Square, No. 2, 300041 Timisoara, Romania; folescu.roxana@umft.ro

**Keywords:** *Populus nigra* L., black poplar, *Populus nigra* L. buds, antioxidant, anti-inflammatory, antimicrobial, antidiabetic, antitumor, vasorelaxant, hepatoprotective, hypouricemic properties, melanin production

## Abstract

*Populus nigra* L. (Salicaceae family) is one of the most popular trees that can be found in deciduous forests. Some particularities that characterize the *Populus* genus refer to the fact that it includes more than 40 species, being widespread especially in Europe and Asia. Many residues, parts of this tree can be used as a bioresource for different extracts as active ingredients in pharmaceuticals next to multiple benefits in many areas of medicine. The present review discusses the latest findings regarding the phytochemical composition and the therapeutic properties of *Populus nigra* L. buds. The vegetal product has been described mainly to contain phenolic compounds (phenols, phenolic acids and phenylpropanoids), terpenoids (mono and sesquiterpenoids), flavones (e.g., apigenol and crysin), flavanones (e.g., pinocembrin and pinostrombin), caffeic/ferulic acids and their derivates, and more than 48 phytocompounds in the essential oils. The resinous exudates present on the buds have been the major plant source used by bees to form propolis. Several studies depicted its antioxidant, anti-inflammatory, antibacterial, antifungal, antidiabetic, antitumor, hepatoprotective, hypouricemic properties and its effects on melanin production. All these lead to the conclusion that black poplar buds are a valuable and important source of bioactive compounds responsible for a wide range of therapeutic uses, being a promising candidate as a complementary and/or alternative source for a large number of health problems. The aim of the review is to gather the existing information and to bring an up to date regarding the phytochemical and therapeutic uses of *Populus nigra* L. buds.

## 1. Introduction

Currently, medicinal plants are playing a vital role in the management of a wide range of both acute and chronic pathologies. Although in the last decades technological development has undergone an impressive evolution, conscious efforts need to be conducted in order to properly identify, recognize and position medicinal plants and to characterize the specific molecular fingerprint of active phytochemicals in the design and implementation of new therapeutic strategies [1].

One of the most widespread trees that can be found in deciduous forests is *Populus nigra* L., which belongs to the Salicaceae family. Many residues, parts of this tree can be used as a bioresource for different extracts as active ingredients in pharmaceuticals, having also multiple other medical applications [2].

The Salicaceae family contains 56 genera from which two intensively studied and reported for a wide range of biological activities: *Populus* and *Salix* [3]. This family includes woody plants, which have alternate leaves. The fruit is a valvicidal capsule and the seeds have hairs; this anatomical aspect helps the species to reproduce because the spread is done through the wind. The flowers are grouped in clusters and have no floral covering [4]. They appear mainly through the northern temperate regions, ranging from North America through Eurasia and Africa. This family has high adaptability that allows them to occupy significant territories, due to common features such as easy dissemination through the wind and high regeneration capacity [2].

Some particularities that characterize the *Populus* genera refer to the fact that it includes more than 40 species, being widespread especially in Europe and Asia. In Romania poplar species grow through wet meadows and depressions, but can be also found in the plains. Black poplar is a tall tree; its height can reach up to 30 m and its diameter up to 2 m. The roots are adherent, the rhytidome is thick, blackish. The buds, which are actually the part of the plant explored in phytotherapy, are large, 2 cm long and 5–8 mm thick, conical, elongated, the tip is sharp and slightly curved, yellow-brown. On the surface, they have viscous resins with a weak balsamic but aromatic smell. On the central axis of the buds there are 4–8 oval and sharp bracts; they are adherent due to the resins in the composition. Additionally, due to the resins, which actually cover the buds, they have a shiny appearance. *Populus* species have a long history in traditional medicine, with uses in many areas [2,4]. The species name “*Populus*” derives from Latin, which means people. In ancient times the poplar trees were often planted around public meeting places, which is a historical explanation for the name of this plant. In Europe, poplar buds appeared for the first time in John Gerard’s book (1597), which described the use of the buds in a healing ointment, with beneficial effects against inflammations [5,6].

In traditional phytotherapy importance was given also to poplar bark, which was assigned with astringent, anti-inflammatory, anti-rheumatic and antiseptic properties [2]. Poplar buds were used as tincture, infusion, powder or ointment [7,8]. The tincture was recommended as remedy in asthma and other respiratory conditions, such as bronchitis, cough, tracheas, laryngitis, sore throat, influenza, gout and pulmonary hemorrhage while the ointment was used in the treatment of dermatological disorders such as ulcerations, hemorrhoids, anal fissures or in gouty and rheumatic inflammation [9]. Moreover, the poplar bark was used topical in superficial skin lesions, sun burns, insect bites or contusions [10].

*Unguentum populeum* is the oldest formulation, which was prepared from poplar buds. It was and continues to be used for its anti-inflammatory and antihemorrhoidal properties [2]. The leaves of this tree are used as a tonic and antiseptic [11]. Black poplar buds are employed for the anti-inflammatory, antipyretic, antiallergic, antimicrobial, antidiabetic and antitumoral properties; moreover, they have beneficial effects against skin infections and antiaging potential [9,12,13].

## 2. Phytochemical Composition of *Populus nigra* L. Buds

**Figure 1:**
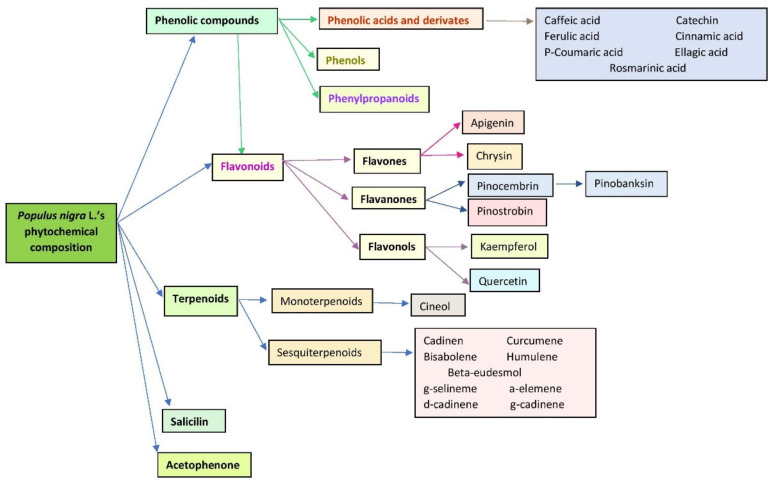
Main classes of phytochemicals in *Populus nigra* L. buds.

*Populus nigra* L. buds have been described mainly to contain phenolic compounds (phenols, phenolic acids, phenylpropanoids and different subgroups of flavonoids) and terpenoids (mono and sesquiterpenoids; Figure 1) [14,15,16,17,18].

It has been suggested that the presence of a large number of flavonoids and phenolic compounds are responsible for most of the biological and pharmacological activities of *Populus nigra* L. buds [7] (Table 1 and Table 2). Literature reports that black poplar buds contain flavones (e.g., apigenol and crysin) and flavanones (e.g., pinocembrin and pinostrombin; Figure 1). Beside this class of phytochemicals, other important representatives are the phenolic compounds, such as caffeic and ferulic acids and their derivatives (Figure 1). Moreover, Jerković et al. described the presence of more than 48 phytocompounds in the essential oils obtained from *Populus nigra* L. buds (Croatia). Cadinen, cineol, curcumene, bisabolene, farnesol, humulene and acetophenone were detected in significant concentration [16]. Isidorov et al. analyzed by GC–MS the chemical composition of hexane and ether extracts from poplar buds (Poland). They have described that the hexane extract contains more than 54 neutral compounds, like 1,8-cineole, salicylaldehyde, cinnamate and the ether extract contain acid compounds such as ferulic acid, caffeic acid, pinocembrin, p-coumaric acid and benzyl-caffeate [19]. They have also mentioned that the main fraction contains PCA and their esters, which give antiseptic properties to the extracts obtained from this vegetal product [20].

Recently, another important study about the chemical composition of this vegetal product was described by Ristivojević et al. This research group identified more than 75 phenolic compounds in poplar bud ethanolic extracts (collected from Serbia). They have reported the presence of phenolic acids and their derivatives (caffeic acid and p-coumaric acid), flavan-3-ols (four diastereoisomers of catechin), flavonols, flavanonols, glycosides (apigenin-7-O-glucoside) and phenolic glycerides [21]. Following the NMR spectroscopy and MS analysis, Dudonne et al. have shown that aqueous black poplar bud extracts (Bulgaria) contain phenolic acids (caffeic, cinnamic, coumaric, ferulic and di-O-methyl caffeic acid), flavonoids (pinocembrin and pinobanksin) and also salicin [22]. The phytochemical screening of methanol, hexane, chloroform and water extracts of black poplar buds (Algeria) has shown that beside flavonoids, tannins and terpenoids the extracts are a rich source of polyphenols, terpenes and alkaloids. Additionally, it was mentioned the absence of anthocyanin, saponins and quinones [23].

Greenway et al. analyzed by GC–MS the chemical composition of twelve *Populus nigra* L. bud exudates collected from seven different countries (United Kingdom, France, Italy, Netherlands, Belgium, Russia and East of China). Results have shown that all the specimens present high levels of caffeic and isoferulic acids and low levels of coumaric and cinnamic acids. The specimen from United Kingdom presents an increased amount of flavones (pinocembrin and pinobanksin), and also a higher amount of chrysin and galangin than the other specimens [14].

Nassima and coworkers identified phenolic compounds in methanolic extracts and ethyl acetate fraction of *Populus nigra* L. buds (Ouzou, Algeria). Both extracts revealed a difference regarding the phenolic composition of the screened samples. Phytocompounds such as myrcitrin, luteolin7-O-glucoside, luteolin and apigenin were found only in ethyl acetate fraction in the amount of 13.42 mg/g of residue, 39.86 mg/g of residue, 10.81 mg/g of residue and respective 1.12 mg/g of residue. Both extracts contained ellagic acid, kaempferol and p-coumaric acid, although the highest concentration was observed for the ethyl acetate fraction. Rosmarinic acid and quercetin were only detected in the methanolic extracts of *Populus nigra* L. buds in the amount of 14.46 mg/g of residue and respective 2.07 mg/g of residue [24].

Rubiolo et al., applied two chromatographic methods (HPLC, GC–MS) for the quantitative determination of phenolic acids and flavonoids, including chrysin, galangin, pinocembrin, pinostrobin and tectochrysin in black poplar bud absolute (produced in Grasse, France). According to HPLC and the GC–MS method, pinostrobin (3.48% and 5.32%), respectively, pinocembrin (2.79% and 3.64%) and chrysin (2.78% and 4.15%) were the most representative compounds in the selected extracts [25].

Studies are trying to use supercritical CO_2_ extraction of flavonoids and volatile compounds, as an environmentally friendly alternative to the ethanol or Soxhlet extraction of *P. nigra* L. buds [26]. The team of Mainar et al. investigated for the first time SC-CO*_2_* extracts of poplar buds (Zaragoza, Spain) using GC–MS chromatography to assess chemical composition. Based on the mass spectra and comparison with Wiley library database, 1-(2, 6-dihydroxy-4-methoxyphenyl)-3-phenyl-(E)-2-propen-1-one was identified as the main compound. Moreover, volatile compounds (beta-eudesmol and alpha-eudesmol) were found to be similar to the results obtained for *Populus nigra* L. essential oil by Jerković et al. [26]. Research conducted by Jerković and Mastelic identified, forty-eight compounds among poplar buds volatiles, using GC–MS. Sesquiterpene alcohols such as β-eudesmol and α-eudesmol were the principal compounds identified (26.3–28.7%). Other major sesquiterpenes were g-selinene (7.6–8.8%), d-cadinene (7.8–8.6%), a-elemene (3.3–5.2%) and g-cadinene (3.9–4.2%) [16].

Since the research of Mainar et al., left the door open for further investigation and optimization of extraction conditions, Kuś et al., applied supercritical fluid extraction with carbon dioxide in a supercritical state and UPLC–DAD analysis of dried poplar buds of *Populus nigra* L. (Poland). The extraction was conducted under different pressure (8.3–33.7 MPa) and temperature (35.8–64.1 °C). Based on the chromatographic analysis, it was found that the extraction efficiency of phenolic acids and flavonoids depends on the process parameters. The largest levels of bioactive phenols in SC-CO_2_ extracts were 1.52, 47.24, 10.25, 79.56, 1.55 and 2.03 mg/g, respectively, for *p-*coumaric acid, pinocembrin, galangin, pinostrobin, pinobanksin and chrysin at the temperature of 60 °C and pressure of 30 MPa [27].

Currently, studies focus on optimal parameters of the extraction process, which should provide the necessary content of biological active substances. According to this fact, Bondar et al., tried to standardize the sum of flavonoids and hydroxycinamic acids derivatives found in ethanolic extracts (96% and 70%) of *Populus nigra* L. buds (Ukraine). Extraction of the phytocompounds was carried out by the method of percolation and the quantitative determination of flavonoids and hydroxycinamic acids derivatives was performed using spectrophotometry. Results showed that the concentration of the extracting and infusion time influence the qualitative composition of the extract. For obtaining an extract rich in hydroxycinnamic acids (57.70%), it is advisable to use ethanol (70%) and for flavonoids (82.60%), it is advisable to use ethanol (96%) [28].

Black poplar buds are rich in phenols, mainly flavonoids and phenylpropanoids and their esters [29]. The resinous exudates present on the buds have been the major plant source used by bees to form propolis. The latter is characterized by similar chemical composition although with some differences [27], for example, the organic compound benzyl benzoate, which is present in the majority of poplar type propolis samples, is not detected in the volatile oils of poplar buds [21]. The work performed by Pavlovic R et al., and published this year regarding new different methods of analytical characterization of propolis samples (a case study) lead to the conclusion that HPLC-Q-Exactive-Orbitrap^®^-MS analysis is an extremely important analytical method for the appropriate identification of several valuable metabolites present in propolis samples and that NMR (nuclear magnetic resonance) could provide additional information concerning isomeric compounds, which should be identified [30].

Propolis is known for the instability of both its composition and biological activity, and recent research approaches suggests that *Populus nigra* L. extracts could be a favorable replacement [31], because it is much easier to manage and standardize their chemical composition, which is crucial for quality control [27].

## 3. Black Poplar Buds’ Properties

### 3.1. Antioxidant Properties

Debbache et al., examined, in vitro the antioxidant effects of poplar bud extracts (Algeria). Seven fractions were used (ethanol, ethyl acetate, aqueous of ethyl acetate, hexane, aqueous of hexane, chloroform and aqueous of chloroform), which were obtained by a selective extraction procedure. Results showed that the aqueous chloroform fraction extract had the highest antioxidant capacity [53].

Wang et al., investigated in vitro (murine macrophage RAW 264.7 cells) and in vivo (animal experimental model on male ICR (Institute of Cancer Research) mice) the antioxidant effects of an ethanolic extract obtained from Chinese poplar buds (*Populus × canadensis*). Results have shown that the extract in different concentrations (10–150 µg/mL in vitro and 25 and 100 mg/kg in vivo) exhibit strong free-radical scavenging activity [8]. Using the modified Folin Ciocalteu and photochemiluminescence methods Stanciu et al. demonstrated that the ethanolic extract of black poplar leaf-buds (Romania) present high antioxidant properties. Results have shown that the antioxidant capacity increased continuously in time from 183.85 to 391.48 nmol/mg dry weight [54].

Mainar et al., evaluated the antioxidant activity of poplar buds (Spain). Using DPPH assay the research group have showed that the ethanolic extract is a powerful source of natural antioxidants, with a radical scavenging potential comparable to the well-known etalon, Trolox [26]. Merghache et al. evaluated the antioxidant properties of black poplar bud hydroalcoholic extracts (Algeria), using different concentrations (5, 10, 25, 50, 75, 100, 250, 500, 750 and 1000 µg/mL). The best antioxidant effect was elicited at the concentration of 1 mg/mL with a potential similar to the antioxidant activity of ascorbic acid [23].

### 3.2. Anti-Inflammatory Properties

**Figure 2:**
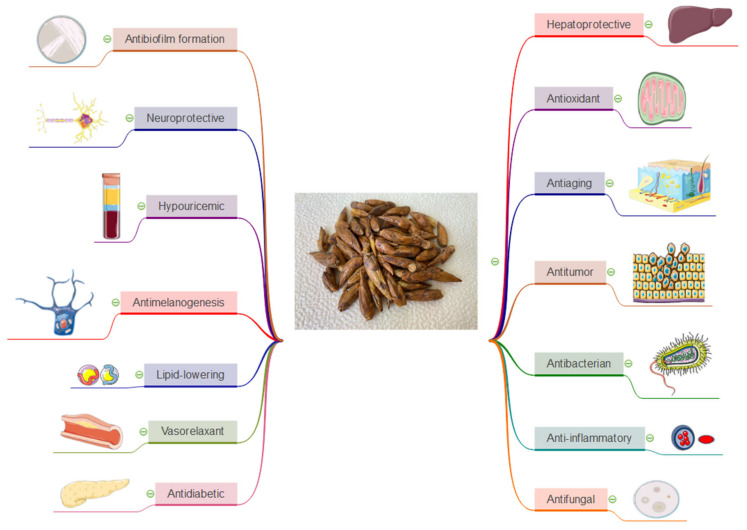
A brief overview of the therapeutic uses of *Populus nigra* L. buds.

In addition to the strong antioxidant effects (Figure 2), poplar buds are being reported especially for their anti-inflammatory properties (Figure 2). This effect could be attributed to the high amount of flavonoids, like quercetin, pinocembrin and also to phenolic acids [22,55].

Dudonne et al., evaluated in vitro the beneficial effects of poplar bud extract on skin aging, using the Oxygen Radical Antioxidant Capacity (ORAC) assay. Extracts (25–200 μg/mL) showed a strong modulation of gene transcription that are involved in inflammatory responses (CCL5 genes) and also cell renewal (KLF10, E2F-4 transcription factor and ZFP36L1) [22]. Colantonio et al. reported that *Populus balsamifera* L. buds (commonly known under the name balsam poplar) can be used to treat wounds and as a pain reliever. Balsam poplar buds are covered with bioactive compounds such as salicylates, which have been reported for antiadipogenic, analgesic and also anti-inflammatory activities [56]. Due to their rich flavonoids components (especially pinocembrin and pinostrombin), poplar bud ethanolic extracts (20–40 µM; Poland) significantly reduced the proinflammatory interleukins, IL-6 and IL1β on an experimental in vitro model using the HGF-1 cell lines [57].

Debbache et al., examined in vivo the anti-inflammatory effect of poplar bud extract (Algeria; water and chloroform extraction). The anti-inflammatory potential was evaluated on an experimental animal carrageenan-induced mice paw edema model. A dose of 200 mg/kg of the poplar bud extract was administered. Results showed a potent anti-inflammatory activity in a similar manner as the reference drug (diclofenac, 50 mg/kg) [53]. Wang et al., analyzed in vivo (animal male ICR mice strain induced with LPS endotoxemia and acute pulmonary damage) the effects on acute inflammatory symptoms of an ethanolic extract obtained from poplar buds (China). Results have shown that the extract in different concentrations (25 and 100 mg/kg) exhibits significant anti-inflammatory effects by inhibiting the production of inflammatory cytokines (IL-6, IL-10 and TNF-α) and blocking the activation of the nuclear factor [8].

Soromou et al., also demonstrated that pinocembrin provides in vitro and in vivo protection against LPS induced inflammation and regulates the production of IL-6, IL-10, IL-1β and TNF-α. In vivo, using a mouse model of LPS induced acute lung injury, 20 or 50 mg/kg pinocembrin attenuates the development of pulmonary edema and histological severities [58].

Using different analytical methods Oancea et al., demonstrated that *Populus nigra* L. buds contain four volatile compounds (betulen, α, β and γ-betulenol, δ-humulen and α-caryophyllene), which have high oxidation–reduction potential. Essential oils in cosmetics have many beneficial effects for skin problems like: help to enhance the tissue tonicity, regenerate and rejuvenate the epidermis [59]. Although there are a few studies focused on the antiaging effects of poplar bud extract, this aforementioned one suggests that the vegetal product is a promising candidate for future skincare formulations.

### 3.3. Antibacterial and Antifungal Effects

Beside the significant antioxidant activity, many phenolic compounds present antibacterial effects (Figure 2) [60]. Gulhan Vardar-Unlu et al. conducted a study regarding the antibacterial activity of poplar bud methanolic extract (central Anatolia, Turkey). They concluded that this extract has a wide spectrum of antibacterial activity, the most sensitive being the Gram-positive bacteria such as *Staphylococcus aureus* (MIC (minimum inhibitory concentration) of 0.50 mg/mL^−1^), *Streptococcus pyogenes* (MIC of 0.50 mg/mL^−1^) and *Enterococcus faecalis* (MIC of 1.00 mg/mL^−1^) [61].

Furthermore, De Marco et al., demonstrated that propolis and poplar bud resins ethanolic extracts from Italy present significant antibacterial potential against *Pseudomonas aeruginosa* (MIC of 125 μg/mL) and inhibit biofilm formation [62]. Benedec et al. have shown that black poplar bud ethanolic extract collected from Romania present strong antioxidant activity due to an increased amount of polyphenolic compounds. Moreover, using the disk-diffusion method, the research group has demonstrated that the ethanol extract of black poplar bud presents in vitro antibacterial activity against Gram-positive bacteria, such as *Staphylococcus aureus* (inhibition zone diameter is 13 mm)*,* and *Listeria monocytogenes* (inhibition zone diameter is 15 mm) [63]. Merghache et al. demonstrated that poplar bud aqueous extract (Algeria) elicited significant antifungal activity against *Candida albicans* (MIC = 45.16 µg/mL) [23].

Debbache et al., tested the potential antifungal activity of 4 types of extracts (ethanol, aqueous ethyl acetate, chloroform and aqueous chloroform) from poplar buds (Province of Amizour, Algeria) on *Aspergillus niger* and *Fusarium polyferatum*. Results evaluated by measuring inhibition zones surrounding plant extracts showed that all screened extracts elicit moderate activity against fungal strains (inhibition zones between 6 and 9 mm) [7].

Different research studies related to this topic indicate that poplar bud extract can be used for the antimicrobial and antibiofilm activity, effects that may be related to the presence of apigenin, pinocembrin kaempferol and other flavonoids and phenolic acids (caffeic and ferulic) [1,24,64]. Using the disk-diffusion method, Nassima et al. showed that 100 µL of poplar bud extract possess antimicrobial effects (diameters ranging from 6.6 to 21.3 mm) and inhibit the biofilm formation of *Staphylococcus aureus* and *Bacillus subtilis* [24].

### 3.4. Antidiabetic Properties

In a comprehensive study, Shiquin Peng et al. evaluated the antidiabetic effects of the ethanolic extract obtained from poplar buds (China). They conclude that the 50% fraction of poplar bud extract increased insulin sensitivity, reduced insulin resistance and decreased the glycated hemoglobin and glycosylated serum proteins, in vivo on an animal experimental model of diabetic mice. Furthermore, they revealed that poplar buds (50 or 100 mg/kg/day for 4 weeks) caused a more than 25% decrease in the serum insulin level of HFD- and STZ-induced T2DM model mice and were more prominent than that of metformin (100 mg/kg/day) (Figure 2). Compared with the control group, treatment decreased MA and increased SOD. It is well known that diabetes can cause different types of inflammation. In this study was also shown that this extract could reduce inflammatory factors, such as IL-6, TNF-α, MCP-1 and COX-2 in liver homogenate [65].

Liu et al., investigated in vitro the effects of pinocembrin, galangin, chrysin and pinobanksin from propolis on insulin resistance and Akt/mTOR signaling using insulin resistant HepG2 cells by an immunoassay. Results showed that only galangin (80 µM) and pinocembrin (4 µM) could increase glucose consumption and glycogen content by enhancing the activities of pyruvate kinase. Moreover, pinocembrin and galangin have elicited synergistic effects on the improvement of insulin resistance [66].

### 3.5. Effects on Melanin Production

Skin possesses highly effective protector mechanisms, such as antioxidant enzymes (superoxide dismutase, catalase and glutathione peroxidase) and non-enzymatic antioxidant molecules (vitamin E, vitamin C, glutathione and ubiquinone) [67]. These protective mechanisms decrease throughout the lifetime and must be supported with high antioxidant products to neutralize reactive oxygen species and also to limit possible inflammation that may occur. An example of a natural product used successfully for this purpose is represented by poplar buds (Figure 2) [68,69,70].

The increase of melanin in the epidermis can cause many disorders such as lentigo and inflammatory pigmentations [71]. Hyperpigmentation is one of the most frequent skin problems due to the influence of hormones; women tend to get hyperpigmentation more frequently than men [72]. The use of depigmenting, whitening products is very common for Caucasian women and there are many studies forward towards this area. A significant number of plant extracts are potent inhibitors of melanin formation with an action comparable to chemical compounds (e.g., hydroquinone) having as an advantage the fact that they are not associated with the cytotoxicity of melanocytes [73]. The group of Maack et al., has evaluated the tyrosinase inhibitory capacity of ethanolic black poplar extract. Results have shown that the absolute extract inhibit in vitro the tyrosinase activity in cultured B16F10 murine melanoma cell lines, reducing melanogenesis [74].

### 3.6. Antitumor Properties

There are not many studies that report the in vitro and/or in vivo antitumor potential of black poplar extract. However, some research has been conducted on pure phytochemicals that can be found in the extracts of poplar buds. One of the most common components in *Populus nigra* L. is pinocembrin. This is a flavonoid found in high concentration in *Populus nigra* L. extracts. Additionally, for chrysine and pinobanksin-3-O-acetate significant values were signaled [55,75].

Studies have shown that this flavanone possesses a vast range of pharmacological effects including antimicrobial, anti-inflammatory, neuroprotective, antioxidant and the ability to reduce reactive oxygen species and regulate apoptosis (Figure 2) [76]. The group of Cheng et al. demonstrated that in the case of LNCaP human prostate cancer cell lines, 100–150 µM pinocembrin presented antiproliferative and proapoptotic properties [77]. Pinocembrin have been shown to present cytotoxicity against HCT116 human colon cancer cell lines. In this case of the phytocompound increased the activity of Caspase 3, Caspase 9 and the mitochondrial membrane potential, but at the same time, it did not affect the cytochrome P450 reductase, quinone reductase and glucuronosyltransferase activity [75]. Gao et al., investigate the effect of different concentrations of pinocembrin (100–200 µM) in vitro on SKOV3 human ovarian cancer cell lines using some consecrated assays (cell scratch assay, Annexin V-FITC/PI staining, Western blot and RT-PCR). Results revealed that this molecule inhibited the proliferation, migration and promoted apoptosis of the tested ovarian cancer cell line [78]. Kumar et al., have demonstrated that pinocembrin (50, 100 and 200 µM) induced apoptosis in HCT116 human colon cancer cell lines. It decreased the MMP with the subsequent release of cytochrome C and increase the level of Caspase 3 and 9 [79].

With respect to this direction the group of Zheng et al., highlighted the antitumor effects of pinocembrin extract against melanoma cells (A375, B16F10) in vitro and in vivo on an experimental model that includes B16F10 cells implanted in C57BL/6 mice strain. The phytocompound, in a dose dependent manner inhibited the proliferation of melanoma cells and suppressed autophagy through the activation of Pl3K/Akt/mTOR pathway. These events have an important role as a dual mechanism responsible for the induction of melanoma cell death. Results also showed that 50–75 mg/kg pinocembrin successfully reduced tumor volume and weight in B16F10-implanted mice [80].

Another major constituent found in the methanolic extract obtained from poplar buds (Gdańsk, Poland) is the flavonoid pinostrobin [57]. Numerous studies performed on this phytocompound have depicted anticancer potential and anti-inflammatory, antiviral, antioxidant, antiprotozoal and antimicrobial capacity [38].

Bail et al. studied the interaction between pinostrobin and the estrogen receptor in the presence or absence of 17β-estradiol (E2) or DHEAS, in a stably transfected human breast cancer cell line (MVLN). They have found an antiaromatase activity (IC_50_ = 10 μM) without decreasing DHEAS- or E2-stimulated cell proliferation and without binding to the estrogen receptor. Since aromatase is responsible for the conversion of testosterone to estrogen, inhibition of the enzyme could reduce estrogen levels, and therefore the probability of hormone-related cancer [81].

Pinostrobin demonstrated a strong antitumor activity on mammary carcinoma cells, which was partially explained by topoisomerase inhibition [82,83,84]. This enzyme is responsible for DNA strand break repair, allowing the broken strand to rotate on the intact one and reducing the torsional tension of the molecule during the process [85].

Jaudan et al. investigated the apoptotic potential of pinostrobin in vitro on cervical cancer cells (HeLa) using various methods of analysis (TUNEL assay, AnnexinV-FITC, AO/EB staining and DCFH-DA staining). The phytocompound demonstrated dose dependent growth inhibition potential. It also reduced GSH at 100 μM and NO_2-_ levels at 50 μM. Apoptosis was induced by Caspase dependent pathways, decreased mitochondrial membrane potential and subsequent increased apoptotic events at 100 μM [86].

Sukradiman et al. have demonstrated pinostrobin’s antitumoral activity on the T47D human breast cancer cell line. Pinostrobin (10, 50 and 100 mg/mL) induced DNA fragmentation and increased the percentage of apoptotic cell. It also increased expression of p53, BAX, Caspase-3 and decreased BCL-2 expression [82].

### 3.7. Hepatoprotective Properties

The first attempt to study the hepatoprotective effect (Figure 2) of ethanolic extract of *Populus nigra* L. buds (Province of Amizour, Algeria) belonged to Debbache-Benaida et al. They studied in vivo on Albino mice, the protective effect of ethanolic extract of *Populus nigra* L. (200 mg/kg) against aluminum-induced hepatic toxicity. Histopathologic analysis of treated liver sections revealed normal hepatic architecture. Moreover, the extract protected almost completely the liver against AlCl3-induced hepatic damage and necrosis. This fact can be explained due to the inhibition of Ca^2+^ influx and/or radical scavenging activity [7].

### 3.8. Vasorelaxant Properties

Debbache-Benaida et al., analyzed in vitro, the potential vascular relaxing ability (Figure 2) of ethanolic extract (10.4–10.1 g/L) of *Populus nigra* L. buds (Province of Amizour, Algeria) using vascular preparation from porcine aorta precontracted with U46619, a synthetic analog of the endoperoxide prostaglandin PGH2. Results showed dose-dependent (10–1 g/L) an increase in the percentage of relaxation, which was comparable in both endothelium-intact (67.74%, IC_50_ = 0.04 mg/mL) and -denuded (72.72%, IC_50_ = 0.075 mg/mL) rings of porcine coronary arteries. The mechanism of vasodilatation of coronary arteries was NO-independent, suggesting that the extract acts directly on smooth muscle, probably impeding the calcium inside the cell [7].

### 3.9. Hypouricemic Properties

Nadjet Debbache-Benaida et al., tested in vivo (animal male albino mice, hyperuricemia induced with potassium oxonate) the effect of poplar bud extract (Algeria) on hyperuricemia. Results indicated that for mice that were treated with the ethanolic extract of *Populus nigra* L. buds, administered orally three times per day in different concentrations ranging from 100 to 400 mg/kg, a significant hypouricemic effect could be detected, compared to mice treated with Allopurinol (Figure 2). At the same time, it was found that 200 mg/kg of poplar bud extract antagonized the toxic effect of AlCl_3_; moreover, the extract restored the pyramidal cells to normal in the cerebral cortex of mice, inducing a neuroprotective effect [87].

Havlik et al. investigated the effects of poplar bud extracts (Czech) in vitro and in vivo. They have evaluated the xanthine oxidase activity in vitro and the antihyperuricemic effects in vivo using an experimental animal model that includes rats. Allopurinol (10 mg/kg) was used as a reference drug. Results have showed that 100–500 mg/kg poplar bud extract significantly inhibited xanthine oxidase activity and also presented hypouricemic effects on the experimental animal model if administered over 3 consecutive days [88,89].

## 4. Cautions and Interactions Regarding Black Poplar Use

It is important to take into account that patients presenting salicylate allergies, and pregnant or lactating women should avoid using products containing salicylate derivates, such as black poplar. Moreover, the ingestion of high doses of black poplar preparations can lead to gastrointestinal problems and tinnitus [11].

Due to the presence of salicylic compounds in *Populus nigra* L., the use of other salicylate preparations should be avoided. Additionally, the coadministration of nonsteroidal anti-inflammatory drugs (NSAIDs) can lead to increased plasma levels of NSAIDs and thus, high toxicity causing especially gastric and renal problems. Association of salicylates with coumarin anticoagulants raises the risk of bleeding. Moreover, their coadministration with hypoglycemic sulfonamides (e.g., glicazide) could enhance the risk of hypoglycemia. Association of salicylates with activated charcoal can decrease the absorption of salicylates, while the coadministration of antacids can decrease the effects of the salicylates. It was found that combining salicylates with uricosuric agents will decrease the uricosuric effect and enhance the uric acid production, while the coadministration with selective serotonin reuptake inhibitors (e.g., escitalopram and fluoxetine) or with tricyclic antidepressants (e.g., amitriptyline) will lead to an increased risk of bleeding. Thus, all these associations need to be avoided. An increased risk of gastrointestinal bleeding was observed when salicylates were taken with alcohol, NSAID or corticosteroid treatment. Moreover, when associated with antihypertensive agents, salicylates will decrease the hypotensive effect, leading to high blood pressure levels [90,91].

The oral administration of preparations containing black poplar has the highest risk of iatrogenic events, compared with the local administration.

## 5. Conclusions

The above presented information led to the conclusion that black poplar buds are a valuable and important source of bioactive compounds responsible for a wide range of therapeutic uses. The modern physicochemical “tools” that allow the characterization and standardization of this extract make *Populus nigra* L. buds a promising candidate as a complementary and/or alternative source for an increased number of health problems.

## Figures and Tables

**Table 1:** Therapeutic potential of the main flavonoids in *Populus nigra* L.

Therapeutic Potential of the Main Flavonoids Present in *Populus nigra* L.						
Apigenin [32]	Chrysin [33]	Pinocembrin [34]	Pinobanksin [35,36,37]	Pinostrobin [38]	Kaempferol [39,40]	Quercetin [41,42]
Anti-inflammatory	Anti-inflammatory	Anti-inflammatory	* obtained by biosynthesis from pinocembrin	Anti-inflammatory	Anti-inflammatory	Anti-inflammatory
Antioxidant	Antioxidant	Antioxidant	Antioxidant	Antioxidant	Antioxidant	Antioxidant
Antimetastatic	Antimetastatic	Antimetastatic	Antimetastatic	Antimetastatic	Antimetastatic	Antimetastatic
Antiangiogenic	Antiobesity	Antiatherosclerotic	Antitrypanosoma	Antiulcer	Vasoprotective	Vasoprotective
Antihypertensive	Antiallergic	Vasodilator		Antidiarrheal	Cardioprotective	Cardioprotective
Anti-diabetic	Antidiabetic	Antiischemic stroke		Antivenom	Antidiabetic	Antidiabetic
Neuroprotective	Neuroprotective	Neuroprotective		Neuroprotective	Neuroprotective	Neuroprotective
Antiviral	Hepatoprotective	Hepatoprotective		Antiviral	Hepatoprotective	Antiischemic
Antibacterial	Reproductive health benefits	Nephroprotective (preventive agent)		Antimalarial	Antiallergic	Antiobesity
		Antibacterial		Antiprotozoal	Anxiolytic	Gastroprotective
		Antifungal		Antitrypanosoma	Analgesic	Anxiolytic
		Antiparasitic			Estrogenic/ Antiestrogenic	Antidepressant
					Antibacterial	Antibacterial
					Antiviral	Antiviral
					Antifungal	
					Antiprotozoal	

**Table 2:** Therapeutic potential of the main phenolic acids in *Populus nigra* L.

Therapeutic Potential of the Main Phenolic Acids Present in *Populus nigra* L.						
Caffeic Acid [43]	Ferulic Acid [44]	P-coumaric Acid [45]	Catechin [46,47]	Cinnamic Acid [48,49]	Ellagic Acid [50,51]	Rosmarinic Acid [52]
Anti-inflammatory	Anti-inflammatory	Anti-inflammatory	Anti-inflammatory	Anti-inflammatory	Anti-inflammatory	Anti-inflammatory
Antioxidant	Antioxidant	Antioxidant	Antioxidant	Antioxidant	Antioxidant	Antioxidant
Antimetastatic	Antimetastatic	Antimetastatic	Antimetastatic	Antimetastatic	Antimetastatic	Antimetastatic
Immunomodulatory	Immunomodulatory	Antidiabetic	Antidiabetic	Photoprotective	Vasoprotective	Vasoprotective
Antiischemic	Photoprotective	Antiplatelet	Antiobesity	Antidiabetic	Cardioprotective	Cardioprotective
Antiatherosclerotic	Cardio-vascular protective	Uricosuric	Antiosteoporotic	Antimelanogenesis	Anti-diabetic	Antidiabetic
Neuroprotective	Neuroprotective	Analgesic	Neuroprotective	Neuroprotective	Neuroprotective	Neuroprotective
Hepatoprotective	Pulmonary protective	Antipyretic	Hepatoprotective	Antiviral	Hepatoprotective	Immunomodulatory
Antiviral	Antimelanogenesis	Antiulcer	Cardio-vascular protective	Antimalarial	Photoprotective	Antiallergic
Antibacterial	Antiaging	Antibacterial	Antifungal	Antituberculosis	Antiobesity	Gastroprotective
Antifungal		Antifungal	Antiviral	Antibacterial	Antihypertensive	Analgesic
		Antiviral	Cytoprotective	Antifungal	Antimelanogenesis	Endocrine/Fertility stimulator
		Anxiolytic		Antiparasitic	Antibacterial	Antibacterial
		Antimelanogenesis			Antiviral	Antiviral
		Anticollagenase			Antimalarial	Antibiofilm formation
					Antituberculosis	Antihelminthic

## Abbreviations

AO	Acridine orange
BAX	Apoptosis regulator
BCL-2	B-cell lymphoma 2
CCL5	chemokines (CC motif) ligand 5
COX-2	Cyclo-Oxygenase Enzyme-2
COX-2	cyclooxygenase-2
DCFH-DA	2′-7′dichlorofluorescin diacetate
DHEAS	dehydroepiandrosterone sulphate
DNA	deoxyribonucleic acid
DPPH	2, 2-diphenyl-1-picrylhydrazyl
E2	17*β*-estradiol
E2F-4	transcription factor
EB =	Ethidium bromide
GSH	Glutathione
GS-MS	Gas chromatography–mass spectrometry
HPLC	High performance liquid chromatography
IC_50_	half maximal inhibitory concentration
IL-6,10, 1β	INTERLEUKIN 1, 6, 1β
KLF10	Krupper-like factor 10
LPS	liposaccharide
MA	malonaldehyde
MCP-1	Monocyte Chemoattractant Protein-1
MCP-1	monocyte chemotactic protein 1
MIC	minimal inhibitory concentration
MMP	mitochondrial membrane potential
MS	mass spectrometry
NMR	nuclear magnetic resonance
NO	nitric oxide
P53	tumor protein
PCA	phenol carboxylic acids
Pl3K/Akt/mTOR	phosphatidylinositol-3-kinase; AKT, protein kinase B; mTOR, mammalian target of rapamycin
RT-PCR	real-time polymerase chain reaction
SC-CO_2_	Supercritical carbon dioxide
SOD	superoxide dismutase
TNF-α	tumor necrosis factor α
TUNEL	TdT-mediated dUTP-biotin nick end labeling
U46619	synthetic analog of the endoperoxide prostaglandin PGH2
UPLC–DAD	High-Performance Liquid Chromatography with Diode-Array Detection
ZFP36L1	EGF response factor 1
